# Effect of exercise on ambulatory supine blood pressure in patients with resistant hypertension and peripheral artery disease with claudication

**DOI:** 10.1007/s11739-025-04166-0

**Published:** 2025-11-04

**Authors:** Nicola Lamberti, Elisabetta Pettenuzzo, Mario Tavani, Giovanni Piva, Lorenzo Caruso, Andrea Baroni, Sofia Straudi, Aaron Thomas Fargion, Roberto Manfredini, Fabio Manfredini

**Affiliations:** 1 Department of Neuroscience and Rehabilitation, University of Ferrara, Ferrara, Italy; 2 PhD program in Environmental Sustainability and Wellbeing, University of Ferrara, Ferrara, Italy; 3 School of Medicine and Surgery, University of Ferrara, Ferrara, Italy; 4 Department of Environmental Sciences and Prevention, University of Ferrara, Ferrara, Italy; 5 Unit of Vascular and Endovascular Surgery, University Hospital of Ferrara, Ferrara, Italy; 6 Clinica Medica Unit, University Hospital of Ferrara, Ferrara, Italy; 7 Department of Medical Sciences, University Hospital of Ferrara, Ferrara, Italy; 8 Program of Vascular Rehabilitation and Exercise Medicine, University Hospital of Ferrara, Ferrara, Italy

**Keywords:** Hypertension, Resistant hypertension, Peripheral artery disease, Exercise, Low-intensity interval training

## Abstract

This retrospective study examines the effects of a 6-month home-based walking low-intensity interval training (LIIT) program on systolic blood pressure (SBP) in a cohort of patients with peripheral artery disease (PAD) and controlled (HT) or resistant hypertension (RH). From a cohort of 1011 PAD patients who completed the 6-month LIIT program, the data of those with hypertension without intercurrent therapy changes were analyzed. LIIT included eight bouts of in-home interval walking (walk:rest ratio of 1:1) at slow speed, progressively increased speed, and was maintained by a metronome. Office blood pressure was measured at baseline and after 5, 12, and 26 weeks by the same operator, with the patient lying in supine position. The ankle‒brachial index (ABI) and performance of the 6-min walking test (6MWD) were also assessed. Program updates were performed during visits, and adherence to exercise was verified and classified into tertiles according to the sessions executed. Among the 793 patients studied, 597 had HT, and 196 had RH. All patients safely completed the LIIT sessions (84 ± 11%). Both subgroups had significantly decreased SBP (RH: − 13 ± 15 mmHg; HT: − 10 ± 14 mmHg; between-group *p* = 0.052). Significant group-per-factor interactions (*p* < 0.001) for the decrease in the SBP were obtained according to adherence to exercise in both the RH and HT groups. The ABI of the worst limb significantly increased (RH: + 0.10 ± 0.13; HT: + 0.09 ± 0.12), as did the 6MWD (RH: + 35 ± 44 m; HT: + 39 ± 45 m). In PAD patients with claudication and hypertension, structured low-intensity in-home exercise, in addition to improving mobility, significantly reduced SBP values in RH patients, reinforcing the effect of medical therapy.

## Introduction

Peripheral artery disease (PAD) of the lower limbs is a growing public health problem that affects more than 110 million people, with an overall burden of disease corresponding to an age-standardized disability-adjusted life years (DALY) rate of 5.9 (3.2–10.8) for males and 4.8 (2.7–8.3) for females [1].

Together with an associated high risk of major adverse events (including myocardial infarction, stroke, revascularization, amputation, and death [1–3]), the reduction in blood flow due to stenoses or occlusion of the arteries of the lower limbs may be responsible for intermittent claudication, ischemic pain, and functional impairment, with a reduction in health-related quality of life [1]. Patients with PAD have decreased walking performance and self-reported physical and mental health-related quality of life [4]. One of the main risk factors contributing to PAD development is high blood pressure [1], with the concomitant presence of PAD and hypertension leading to worse outcomes [3–5]. In a cohort study of 4.2 million adults, a 20 mmHg higher than usual systolic blood pressure was associated with a 63% higher risk of PAD [6]. This PAD subgroup has an increased risk of chronic kidney disease, followed by ischemic heart disease, heart failure, atrial fibrillation, and stroke [6].

The optimal management of hypertension in PAD patients needs careful evaluation on the basis of shared risk factors and the specific needs of each condition [5]. Interestingly, in PAD patients, rather than those with a history of hypertension, the presence of out-of-target low and/or high systolic blood pressure (SBP) values is associated with a greater risk for major cardiovascular events [7, 8]. These observations call for optimal blood pressure targets for PAD patients.

In this complex balancing game, the problem of resistant hypertension (RH), which affects approximately 10–20% of treated hypertensive patients and up to 30% of patients in the presence of pseudoresistant conditions [9], needs to be considered [10, 11]. Resistant hypertension is defined as off-target blood pressure despite the use of three different classes of antihypertensive drugs, e.g., a thiazide diuretic, a renin‒angiotensin‒aldosterone system inhibitor, and a long-acting calcium channel blocker at optimized and tolerable doses for more than 3–6 months [10, 12]. In addition, patients with RH should be encouraged to maintain a healthy lifestyle, which includes salt restriction, weight reduction, cessation of smoking, and physical activity or exercise [10, 11], in the presence of advanced chronic and end-stage kidney disease, where renal function decreases twice as fast with RH compared with non-RH [13, 14].

The favorable effects of exercise on blood pressure have long been known [15–18]. In addition, PAD patients, who are often also affected by hypertension, could receive further benefits from exercise [19], but in the presence of claudication, they could lose this potential synergic effect with medical therapy. Unfortunately, little information is available on exercise in PAD patients with RH.

The test-in-train-out low-intensity interval training (TiTo-LIIT) program prescribed at the hospital and performed inside the home was designed for PAD patients [20]. The program, which is based on a few minutes of walking exercise performed in the absence of pain in addition to functional improvements, has been shown to reduce systolic and diastolic blood pressure values and increase ankle–arm index values in a cohort of PAD patients [20–24]. Interestingly, the same program was also found to induce hemodynamic improvements in the upper limbs [25].

The aim of this study in a cohort of PAD patients enrolled in the TiTo-LIIT program was to determine whether, in RH patients, resistance to exercise is also observed in terms of SBP values compared with those in HT patients or if a comparable effect is present.

## Methods

This single-center cohort study retrospectively analyzed a prospectively collected database of PAD patients who were referred after PAD diagnosis to the Unit of Vascular Surgery to the Vascular Rehabilitation Program at the University Hospital of Ferrara.

Between January 2015 and December 2023, a total of 1011 consecutive patients with PAD at Fontaine’s stage II were enrolled in the study program. The local ethics committee (comitato etico area vasta emilia centrale) approved the study (number Oss/277/19). Written informed consent was waived for the nature of the study for the patients who were no longer attending the program. The other patients provided written informed consent.

The study is reported according to the STROBE guidelines.

### Inclusion criteria

For this subanalysis, a cohort of consecutive patients with previously diagnosed hypertension according to the ESC criteria [9] was considered. For the final analyses, patients who dropped out of the exercise program and/or who experienced variations in antihypertensive pharmacological therapy during the follow-up were excluded.

### Intervention

All patients received the TiTo-LIIT program [20, 21, 24], which was prescribed at the hospital during approximately monthly visits and performed at home. The program consisted of two daily 10-min sessions of intermittent walking (walk:rest ratio, 1:1) for 6 days per week at a prescribed speed, which was slower than the individual’s walking speed at the beginning and increased weekly. The training speed was converted to a walking cadence (steps/min) and was paced at home via a metronome or a smartphone application. A daily log to be completed after each training session was given to the patients and collected at each subsequent visit. When possible, the training execution was also certified by a caregiver. Adherence to the exercise was calculated as the percentage of certified executed home-walking sessions with respect to the prescribed sessions.

### Outcomes

The study variables were collected at baseline and at every hospital visit (entrance or T0; after 5 ± 1 weeks, T1; after 11 ± 2 weeks, T2; after 19 ± 2 weeks, T3) by the same skilled operators in a temperature-controlled environment in the morning between 9:00 and 12:00 AM. The same time of measurement was used for each patient throughout the entire observation.

### Primary outcome

The primary outcome for the study was the measurement of systolic blood pressure (SBP) in the supine position during the execution of the ankle‒brachial index (ABI) measurement according to the guidelines [26]. The patients lay in the supine position on a bed for at least five minutes, and blood pressure was measured at both the anterior tibial and dorsalis pedis arteries. Thereafter, both systolic and diastolic blood pressures were measured three consecutive times by the same experienced medical doctor using a stethoscope (Littmann, 3 M, Saint paul, USA) and a standard blood pressure cuff (Heine gamma G5, Gilching, Germany). Both SBP and DBP were measured in both arms, and those in the higher arm were recorded.

### Other parameters assessed

The participants’ demographics, cardiovascular risk factors and comorbidities, and drug therapy data were collected from the medical documentation of the patients and verified through the online management system of the university hospital of ferrara.

Moreover, the ABI of both limbs (calculated as the ratio between higher ankle pressure and SBP) and DBP were recorded as previously reported.

Finally, an aboveground 6-min walk test was conducted according to the guidelines. Patients were asked to walk back and forth along a 20-m corridor to cover the longest distance possible. The total distance covered (6-min walking distance, 6MWD) and the distance at the onset of the claudication symptoms (pain-free walking distance, PFWD) were also recorded.

### Variable definitions

The overall population was then divided into two subgroups, those with hypertension (HT) and those with resistant hypertension (RH) previously diagnosed, defined as those whose blood pressure remains elevated despite the use of three or more antihypertensive medications, including a diuretic, at maximally tolerated doses [27]. In this subgroup (RH) were included also patients with acceptable blood pressure values, but only with the use of four or more antihypertensive medications. To determine a population of potential nonresponders to variations in SBP after a chronic exercise program, a cutoff value of a decrease in SBP equal to or greater than 5 mmHg, which may be considered clinically significant, particularly in the context of reducing cardiovascular risk [28], was applied.

Adherence to the program was subdivided into three tertiles (excellent, high, moderate) according to the data distribution. Seasonality was divided into spring–summer (for exercise programs that started from February to July) and fall–winter (training starting between august and january).

### Statistical analysis

The data distribution was verified via the Kolmogorov‒Smirnov test. Overtime comparisons of all variables were performed through a repeated-measures analysis of variance or a Freidman test according to the data distribution. The variations between each time point were verified by a paired-samples wilcoxon test.

Two-way analysis of variance (factors: time and variable of interest) was employed to define the factors related to different responses of blood pressure over time. Multiple regression models with an Enter method of selection were used to identify the variables related to the changes in SBP in the whole population and the two subgroups. Moreover, logistic regression models with a stepwise method of selection were employed to determine the impact of participants’ characteristics on the negative response of blood pressure to chronic training. Correlations between variables were verified with Spearman’s rho. Finally, to account for baseline imbalances between subgroups (HT versus RH) a propensity score-matched analyses was performed to obtain balanced group at baseline. No missing data were present in the dataset. A *p* value < 0.05 was considered significant. Statistical analyses were performed with MedCalc^®^ Statistical Software version 23.2.6 (MedCalc Software Ltd., Ostend, Belgium).

## Results

**Fig. 1:**
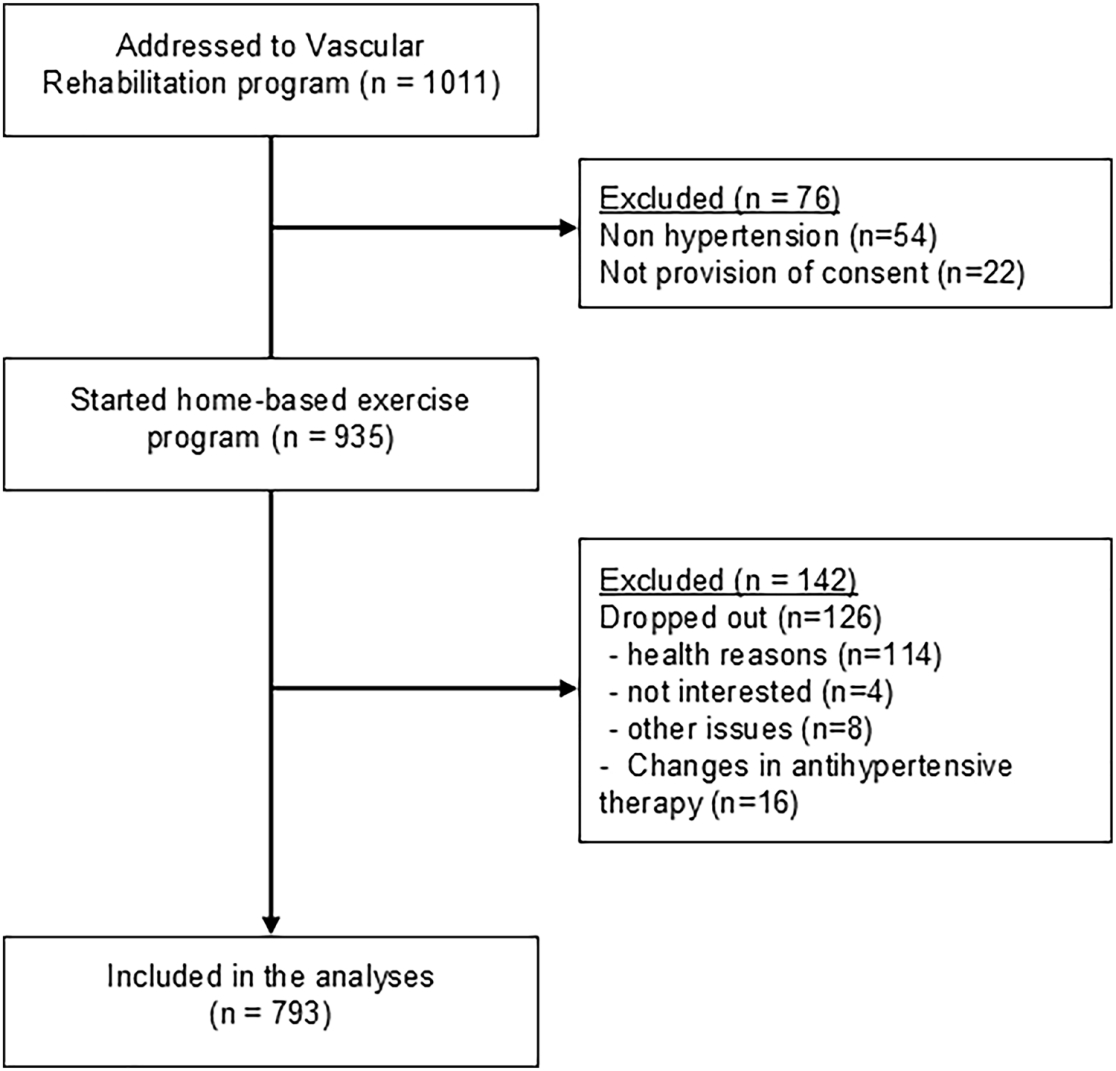
Study flow diagram

From a total of 1011 consecutive patients admitted to the Vascular Rehabilitation Program at the University Hospital of Ferrara, a final sample of 793 patients was analyzed. The reasons for exclusion are reported in Fig. 1.

The final population was composed of 597 people with HT and 196 people with RH. People with RH had significantly more comorbidities and, as expected, greater use of antihypertensive drug therapy, with the exception of angiotensin-converting enzyme (ACE) inhibitors. No differences between the two subgroups were observed in terms of PAD history, severity, or physical performance.

The characteristics of the entire sample and of the two subgroups are reported in Table 1.

**Table 1:** Characteristics of the population enrolled in the study

	Total (*n* = 793)	HT (*n* = 597)	RH (*n* = 196)	Between groups *p* value
Age, years	73 ± 8	73 ± 8	73 ± 7	0.650
Male sex, *n *(%)	601 (76)	441 (74)	160 (82)	0.028
Risk factors				
Smoking	679 (86)	507 (85)	172 (88)	0.541
Hyperlipidemia	607 (77)	438 (73)	169 (86)	0.002
Diabetes	365 (46)	253 (42)	112 (57)	< 0.001
Chronic kidney disease	199 (25)	129 (22)	70 (36)	< 0.001
Comorbidities				
Ischemic heart disease	197 (25)	125 (21)	72 (37)	< 0.001
Stroke/TIA	73 (9)	55 (9)	18 (9)	0.848
COPD	64 (8)	49 (8)	15 (7)	0.828
Cancer	221 (28)	162 (27)	59 (30)	0.422
Rheumatic disorder	60 (7)	47 (8)	13 (7)	0.569
Charlson comorbidity index	7.0 ± 2.2	6.7 ± 2.0	7.6 ± 2.3	< 0.001
Peripheral artery disease				
Years of claudication	4 ± 5	4 ± 5	4 ± 5	0.637
Previous revascularization	196 (25)	160 (26)	36 (19)	0.731
ABI, worst limb	0.62 ± 0.19	0.63 ± 0.19	0.60 ± 0.20	0.063
ABI, best limb	0.84 ± 0.20	0.85 ± 0.19	0.82 ± 0.20	0.057
6-min walking distance, m	294 ± 95	294 ± 97	293 ± 89	0.866
Pain-free walking distance, m	141 ± 95	140 ± 94	144 ± 95	0.599
Antihypertensive drug therapy				
Calcium channel blockers	470	317 (53)	153 (78)	< 0.001
Angiotensin-converting enzyme (ACE) inhibitors	601	447 (75)	154 (79)	0.256
Beta-blockers	552	381 (64)	171 (87)	< 0.001
Angiotensin-II-receptor antagonists	495	329 (55)	166 (85)	< 0.001
Diuretics	418	258 (43)	160 (82)	< 0.001
Others	488	333 (56)	155 (79)	< 0.001
Blood pressure				
Systolic blood pressure, mmHg	157 ± 21	155 ± 22	164 ± 18	< 0.001
Diastolic blood pressure, mmHg	76 ± 10	76 ± 10	76 ± 9	0.931

### Exercise training

The population that completed the exercise training exhibited adherence to the program by completing 84 ± 11% of the prescribed sessions, without any significant difference between the two subgroups (HT: 84 ± 12%; RH: 85 ± 10%; *p* = 0.66). No falls or adverse effects during training execution were reported by the patients.

### Primary outcome

In the entire population, a significant decrease in SBP was observed throughout the time points of collection, with a final variation of − 11 ± 16 mmHg, with significant differences recorded between each period.

A superimposable trend was noted in the two subgroups, with subjects with RH exhibiting a greater decline in SBP (− 13 ± 15 mmHg) than HT participants did (− 10 ± 14 mmHg), even though the group per factor interaction was just nonsignificant (*p* = 0.052). The data are reported in Table 2. Individual changes in SBP are graphically represented in Supplementary Fig S1.

**Table 2:** Values of systolic and diastolic blood pressure in the entire population and in the two subgroups over time

	T0	T1	T2	T3	*T* for trend
Systolic blood pressure (mmHg)					
Entire population (*n* = 793)	157 (156–159)	150^*^ (149–151)	148^*†^ (146–149)	147^*†‡^ (145–148)	*t* = − 18.1 *p* < 0.001
Hypertension (*n* = 597)	155 (154–157)	149^*^ (147–150)	146^*†^ (145–148)	145^*†‡^ (143–146)	*t* = − 15.0 *p* < 0.001
Resistant hypertension (*n* = 196)	164 (161–166)	154^*^ (152–156)	152^*†^ (149–154)	151^*†‡^ (149–154)	*t* = − 10.3 *p* < 0.001
Diastolic blood pressure (mmHg)					
Entire population (*n* = 793)	76 (75–76)	74^*^ (73–75)	73^*^ (73–74)	73^*^ (73–74)	*t* = − 7.1 *p* < 0.001
Hypertension (*n* = 597)	76 (75–76)	74^*^ (73–75)	73^*^ (73–74)	73^*^ (73–74)	*t* = − 6.5 *p* < 0.001
Resistant hypertension (*n* = 196)	76 (73–77)	74^*^ (73–75)	73^*^ (72–75)	74^*^ (73–75)	*t* = − 3.0 *p* = 0.003

*T0* baseline, *T1* after 5 ± 1 weeks, *T2* after 11 ± 2 weeks, *T3* after 19 ± 2 weeks

^*^p < 0.05 with respect to T0

^†^*p* < 0.05 with respect to T1

^‡^*p* < 0.05 with respect to T2

A similar trend was observed for diastolic blood pressure, with a mean significant variation of − 2 ± 9 mmHg (*p* < 0.001) in the whole population and in the two subgroups, without any between-group differences (Table 2).

### Parameters related to blood pressure variations

In both the whole population and the two subgroups, we analyzed whether any parameters may have affected the response of SBP over time.

**Fig. 2:**
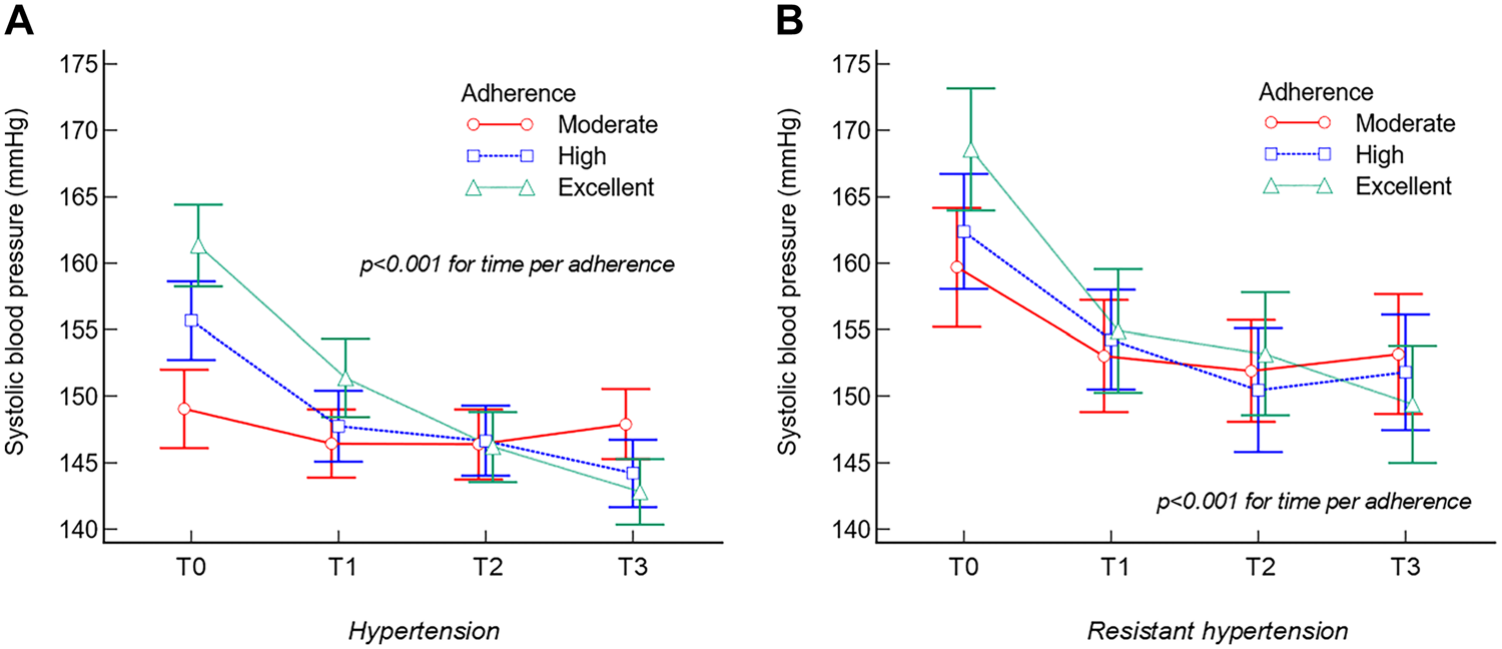
Variations in the systolic blood pressure in the two subgroups according to adherence to the program

In the HT subgroup, the only significant group per factor interaction (*p* < 0.001) observed was between the tertiles of adherence. The subgroup with excellent adherence (from 90 to 100%) presented a decrease in the SBP of − 19 ± 15 mmHg, whereas the subgroup with high adherence (from 81 to 89%) presented a variation of − 11 ± 11 mmHg, which was also significantly greater than that of the group with moderate adherence (from 55 to 80%) at − 1 ± 16 mmHg (Fig. 2A).

Nonsignificant group-by-factor interactions were observed for sex (*p* = 0.731), smoking habit (*p* = 0.343), diabetes (*p* = 0.267), CKD (*p* = 0.602), ischemic heart disease (*p* = 0.074), stroke (*p* = 0.960), COPD (*p* = 0.461), previous revascularizations (*p* = 0.185), and seasonality (*p* = 0.144). The trends are graphically reported in Supplementary Fig S2.

A superimposable trend was observed in the RH subgroup, with adherence acting as the only significant factor (*p* < 0.001), with the subgroup with excellent adherence (from 90 to 100%) exhibiting significantly greater variation (− 19 ± 14 mmHg) than both the high-adherence subgroup (− 11 ± 10 mmHg) and the moderate-adherence subgroup (− 7 ± 19 mmHg) (Fig. 2B).

Nonsignificant group-by-factor interactions were observed for sex (*p* = 0.192), smoking habit (*p* = 0.347), diabetes (*p* = 0.084), CKD (*p* = 0.366), ischemic heart disease (*p* = 0.820), stroke (*p* = 0.139), COPD (*p* = 0.510), previous revascularizations (*p* = 0.133) and seasonality (*p* = 0.804). The trends are graphically reported in Supplementary Fig S2.

According to a propensity score-matched analyses, a subsample of 196 patients with HT was obtained and compared at baseline with RH subgroup (Supplementary table T1). In this subsample, the patients at excellent adherence presented a decrease in the SBP of − 18 ± 14 mmHg, those at high adherence of − 11 ± 10 mmHg, and those at moderate adherence of − 2 ± 14 mmHg (*p* < 0.001). No other significant group-by-factor interactions were noted for sex (*p* = 0.876), smoking habit (*p* = 0.231), diabetes (*p* = 0.554), CKD (*p* = 0.638), ischemic heart disease (*p* = 0.115), stroke (*p* = 0.886), COPD (*p* = 0.440), previous revascularizations (*p* = 0.270), and seasonality (*p* = 0.534).

### Variables related to blood pressure changes

In the entire population, a significant regression model was obtained (*R*^2^ = 0.403; *p* < 0.001) highlighting as significant variables the adherence (in terms of percentage of completed exercise sessions, partial *r* = 0.344) and the baseline SBP value (partial *r* = 0.529). Similar results were obtained in the HT subgroup (*R*^2^ = 0.426; *p* < 0.001) and RH subgroup (*R*^2^ = 0.294; *p* < 0.001). Values are reported in Supplementary Table T2.

### Nonresponders to blood pressure changes after the exercise program

A total of 231 patients at the end of the program with respect to the baseline showed a variation in SBP from − 4 mmHg to higher numbers and were therefore labeled nonresponders.

In the whole population, a significant regression model was noted (*R*^2^ = 0.53, *p* < 0.001), with adherence to the program (odds ratio 4.33) and lower systolic blood pressure at entry (odds ratio 1.06) as the only factors that were retained in the model.

The same two factors were retained in the models created for the HT (*R*^2^ = 0.51, *p* < 0.001) and RH subgroups (*R*^2^ = 0.57, *p* < 0.001).

### Subanalyses in patients with CKD and diabetes

In the entire population, a total of 365 patients were affected by diabetes, 199 by CKD, and 120 had concomitant presence of the two conditions (Table 1).

In the diabetic population, a significant decrease in SBP was observed, with mean values of − 10 ± 11 mmHg in the HT subgroup, and of − 11 ± 12 in the RH subgroup, without any between subgroup difference (Supplementary Fig S3). In relation to the adherence to LIIT, a significant group per factor interaction was observed (*p* < 0.001), with patients with excellent adherence exhibiting a greater decrease (− 19 ± 14 mmHg), with respect to those with high adherence (− 10 ± 12 mmHg) and to those with moderate adherence (0 ± 13 mmHg). (Supplementary Fig S4).

A superimposable trend was observed in the CKD population, with a mean decrease in SBP of − 8 ± 12 mmHg in the HT subgroup, and of − 9 ± 11 in the RH subgroup, without any between subgroup difference (Supplementary Fig S5). In relation to the adherence to LIIT, a significant group per factor interaction was observed (*p* < 0.001), with patients with excellent adherence exhibiting a greater decrease (− 17 ± 11 mmHg), with respect to with high adherence (− 10 ± 12 mmHg) and those with moderate adherence (0 ± 14 mmHg) (Supplementary Fig S6).

### Secondary outcomes

Throughout the exercise program, favorable variations were observed in the outcomes of both the HT subgroup and the RH subgroup. In particular, the former exhibited an increase in the 6MWD of 39 ± 45 m (*p* < 0.001) and in the ABI of the more severe limb of 0.09 ± 0.12 (*p* < 0.001). Superimposable variations were noted in the RH subgroups, with 6MWD variations of 35 ± 44 m (*p* < 0.001) and an improvement in the ABI of 0.10 ± 0.13 (*p* < 0.001).

No between-group differences in changes were observed.

No significant correlations were detected between the variations in SBP and the 6MWD for either subgroup (Supplementary Fig S7).

## Discussion

The study revealed that in a cohort of patients with PAD and hypertension, a low-intensity interval walking program performed inside the home was associated with a reduction in both the systolic and diastolic blood pressure values. The significant decrease in SBP observed in PAD patients with RH was comparable to the reduction in values recorded in the PAD population with controlled hypertension.

Various aspects need to be considered in the discussion, and first, the literature on the effects of exercise in RH in the presence of PAD is very limited.

An analysis of the magnitude of the response in our observational study revealed that the average drop in ambulatory blood pressure with the same treatment was − 10 mmHg for SBP and − 3 mmHg for DBP, without any differences between the RH and HT subgroups.

A similar value was reported in a randomized study in PAD patients not categorized as hypertensive, with a decrease in systolic and mean blood pressures (− 10 ± 3 and − 5 ± 2 mmHg, respectively) after training [29].

In another study in a PAD population, if no changes in SBP were observed after interval walking training, decreased ambulatory pressure variability was reported, with a favorable impact on cardiovascular risk [30].

To compare the results of the present study with the response of BP to exercise, we may refer to a meta-analysis reporting the effects on resting blood pressure after different types of exercise programs to establish optimal exercise prescription practices in the management of resting arterial blood pressure [28]. In this study, involving adults with no predetermined limitations on health or disease state in representation of the general population, the authors analyzed 270 studies and more than 15,000 participants with a focus on SBP. An overall reduction of 4.49 mmHg was reported, with a reduction ranging from 6.8 mmHg for cycling and running to 2.85 mmHg for walking, with a significant reduction in resting SBP following all exercise modes except aerobic interval training [31]. Interestingly, our observational data in a special PAD population with RH revealed a greater reduction following low-intensity aerobic interval training for SBP, with coherent variations with the literature regarding DPB (overall reduction reported of 2.53 mmHg) [31].

Moving on to studies involving patients with RH, in a trial including 140 subjects, a 4-month structured program of diet and exercise delivered in a cardiac rehabilitation setting significantly reduced clinic and ambulatory BP to a greater extent in the experimental group (–12.5 mmHg) than in the usual care group [17]. Similar values were obtained in another study encompassing a multicomponent lifestyle modification [32] or in another randomized trial focusing on aerobic exercise only [33]. Interestingly, together with decreased blood pressure, reduced perceived stress and irritability were observed, as were improvements in psychological functioning [17, 34].

In a systematic review on physical exercise and RH [18], the authors reported that exercise training interventions that combine aerobic and strengthening exercises, which last for at least 8 weeks, decrease both “office” and ambulatory BP measures [18]. In that meta-analysis [18], three randomized trials revealed a reduction in daytime ambulatory BP of − 11.7 mmHg after exercise training. These data support the concept that exercise represents an effective option to reduce BP in patients with RH, even though these studies were conducted involving mainly nondiseased community-dwelling older adults, all aged 60 years or younger, and, in some cases, excluded PAD patients [18]. However, the improvements in SBP are not far from those reported in the present retrospective analysis in a PAD population with restricted mobility and a mean age of 72 years.

Other aspects, in addition to the simultaneous presence of RH and PAD, differentiate our data from those of other exercise studies. In the available literature, the populations generally include relatively young subjects enrolled in programs under supervision, a combination of factors that make it possible to safely conduct even high-intensity training programs. The problem of acting on widespread pathologies and conditions would require the identification of sustainable and widely spread intervention models. The data presented here refer to a program for PAD patients involving low-intensity in-home exercise that can be administered to every person with at least 10 m walking autonomy, including assisted walking, with possible enrollment of severe PAD patients or those with various comorbidities. Interestingly, in the short term, in patients with RH, light-intensity exercise was effective at lowering both the systolic and diastolic pressures during the 10-h daytime, unlike moderate-intensity exercise and forearm blood flow. In a recent randomized study, moderate-intensity exercise, such as high-intensity interval exercise, reduced clinical DBP immediately postexercise in RH patients [35]. With respect to the effects of LIIT, functional benefits at discharge are greater than those observed after pain walking are reported, as are favorable long-term outcomes [20, 23, 24, 36]. This training model also allows the transfer of the model with benefits to other patient populations with limited mobility, such as patients with end-stage kidney disease, stroke, or multiple sclerosis [37–41].

In the present study, the issues of high eligibility and home execution were critical factors in obtaining patient adherence. Indeed, adherence is a critical factor, considering that in a multicomponent lifestyle program in patients with RH, a 12% attrition rate was reported [32] and that compliance with the TiTo-LIIT program was the only factor associated with SBP reduction. These data were associated with a functional recovery of 38 m in 6 min, which is consistent with the minimal clinically important difference [42] and with a recovery of mobility and cardiovascular fitness.

Another difference from the available literature, in this case specifically targeting PAD patients, is the maintenance of generally stable or increased peripheral blood pressure values after the TiTo-LIIT program despite the drop in systolic pressure upstream. This dual hemodynamic benefit, which may favor the hemodynamic status and at the same time limit the risk of ischemic suffering in lower districts, is an aspect already observed by our research group and is not commonly reported in the literature to our knowledge [20, 22, 25]. A positive impact of the proposed type of exercise on endothelial function and nitric oxide production, an action on the sympathetic system and on the arterial stiffness pillars [12], may explain these changes and a more favorable hemodynamic picture in the lower and upper limbs after the TiTo-LIIT program [22, 25, 43].

Finally, a relevant aspect of this study worthy of discussion is the use of supine systolic pressure as an outcome measure. We have therefore deviated from the guidelines [44] in terms of posture, which call for a subject sitting with his or her back supported by a chair, with his or her legs uncrossed, feet flat on the floor, and a bare arm resting on the table. The impact of posture on measurement has been studied [45], with different results in various trials showing lower SBP values after the supine position than after the sitting position [46, 47] or, conversely, higher values [48]. The change in posture can cause hemodynamic changes with variability between supine or sitting values depending on conditions related to the measurement, such as the duration of the rest period, which decreases in the course of relaxed lying, or patient status, including age and body mass index, influencing these differences [46, 47]. A paper highlighted the difference in values in diabetic patients when the World Health Organization recommended the equivalence of sitting and supine BP readings [49]. Interestingly, in terms of global risk, the supine position BP measurement predicted all-cause and cardiovascular mortality better than BP measured in other postures did [50]. Moreover, it was recently reported that supine HTs, regardless of seated HTs, are associated with greater cardiovascular risk than seated HTs alone [51]. In our study, from a methodological point of view, we opted to perform only one supine measurement to obtain a standardized resting blood pressure value in a combined ankle‒arm index measurement session. This fact allowed us to assess the exact differential systemic and peripheral values for the abovementioned clinical reasons in terms of general and local risks [46, 47]. Furthermore, only one type of measurement was used to avoid confounding discrepant information to the patient.

This study has several limitations. First, this was a retrospective study even if data were prospectively collected in a unique center and in the same setting by the same operators who were not blinded to the measurement, and data were reported and signed daily in the medical records. Some transcription errors cannot be excluded. Second, the aforementioned use of supine systolic pressure may represent a limitation compared with the absolute values reported in the literature; however, it does not affect the data of the individual variations, which are generally comparable.

In conclusion, resistance to the combination of antihypertensive drugs does not correspond to resistance to exercise, considering that the lower effect observed is related mainly to adherence to the prescribed TiTo-LIIT program.

If exercise and medical therapy are combined, they increase the antihypertensive effects of pharmacological therapy alone [52], and this is generally also true in the presence of a condition that severely limits mobility, such as vascular claudication, and in the presence of polytherapy in RH.

In the absence of trials conducted in patients with PAD and RH and considering that the available studies are carried out on in younger subjects, this real-world study derived from a clinical setting may add useful information. Future randomized clinical trials are needed.

## Data Availability

The datasets generated during and analyzed during the current study are available from the corresponding author on reasonable request.
